# Impact of parturition on maternal cardiovascular and neuronal integrity in a high risk cohort – a prospective cohort study

**DOI:** 10.1186/s12884-019-2570-6

**Published:** 2019-11-05

**Authors:** Katrina Suzanne Evers, Evelyn Annegret Huhn, Sotirios Fouzas, Christian Barro, Jens Kuhle, Urs Fisch, Luca Bernasconi, Olav Lapaire, Sven Wellmann

**Affiliations:** 1 Division of Neonatology, University Children’s Hospital Basel (UKBB), Spitalstrasse 33, 4056 Basel, Switzerland; 2 Division of Obstetrics and Gynecology, University Hospital Basel, Basel, Switzerland; 3 Paediatric Respiratory Unit and Department of Neonatology, University Hospital of Patras, Patras, Greece; 4 Neurologic Clinic and Policlinic, Departments of Medicine, Biomedicine and Clinical Research, University Hospital Basel, University of Basel, Basel, Switzerland; 5 Institute of Laboratory Medicine, Kantonsspital Aarau, Aarau, Switzerland; 6 Division of Neonatology, University Children’s Hospital Regensburg (KUNO), University of Regensburg, Regensburg, Germany

**Keywords:** Parturition, Brain, Delivery, Pregnancy, Birth, Surrogate marker

## Abstract

**Background:**

To better understand the profound multisystem changes in maternal physiology triggered by parturition, in particular in the underexplored neuronal system, by deploying a panel of pre- vs post-delivery maternal serum biomarkers, most notably the neuronal cytoskeleton constituent neurofilament light chain (NfL). This promising fluid biomarker is not only increasingly applied to investigate disease progression in numerous brain diseases, particularly in proteopathies, but also in detection of traumatic brain injury or monitoring neuroaxonal injury after ischemic stroke.

**Methods:**

The study was nested within a prospective cohort study of pregnant women at risk of developing preeclampsia at the University Hospital of Basel. Paired ante- and postpartum levels of progesterone, soluble fms-like tyrosine kinase-1 (sFlt-1), placental growth factor (PlGF), mid-regional pro-atrial natriuretic peptide (MR-proANP), copeptin (CT-proAVP), and NfL were measured in 56 women with complete clinical data.

**Results:**

Placental delivery significantly decreased all placental markers: progesterone 4.5-fold, PlGF 2.2-fold, and sFlt-1 1.7-fold. Copeptin and MR-proANP increased slightly (1.4- and 1.2-fold, respectively). Unexpectedly, NfL levels (median [interquartile range]) increased significantly post-partum: 49.4 (34.7–77.8) vs 27.7 (16.7–31.4) pg/ml *(p* < 0.0001). Antepartum NfL was the sole independent predictor of NfL peri-partum change; mode of delivery, duration of labor, clinical characteristics and other biomarkers were all unrelated. Antepartum NfL levels were themselves independently predicted only by maternal age.

**Conclusions:**

Parturition per se increases maternal serum NfL levels, suggesting a possible impact of parturition on maternal neuronal integrity.

## Background

Parturition triggers major multisystem changes, most notably hormonal and cardiovascular, which are as precipitate as those in pregnancy are progressive. However, we know little about the impact of parturition and pregnancy on maternal neuronal integrity. Despite some studies of neural change, including effects on brain size [1], neuronal morphology [2] and neuroplasticity [3], postpartum changes in the levels of specific biomarkers for maternal neuronal injury, stress and hemodynamics have not been systematically explored to date.

The scaffold of neurons is composed of certain proteins, including neurofilaments (Nf), which are highly specific major neuronal scaffolding proteins and which are composed by 4 subunits: the triplet of Nf light chain (NfL), Nf medium chain, and Nf heavy chain (NfH), and α-internexin in the central nervous system (CNS), or peripherin in the peripheral nervous system [4]. Neuronal damage, acute or chronic, leads to a release of Nf fragments into the extracellular fluid, cerebrospinal fluid (CSF) and peripheral blood [4–6]. Highly sensitive single molecule array (Simoa) immunoassay has improved NfL detection, particularly in peripheral blood, making NfL a promising and readily accessible biomarker for neuroaxonal injury even in very slowly progressing diseases such as Alzheimer’s disease and before the onset of clinical symptoms [7].

Copeptin, a peptide derived from the same precursor as arginine vasopressin, is a robust and equally accessible biomarker for fluid equilibrium, vascular tone, and individual stress [8–10]. Mid-regional pro-atrial natriuretic peptide (MR-proANP), a stable by-product of atrial natriuretic peptide, is an established biomarker for hemodynamic stress and hypertension [11]. Placental growth factor (PlGF) and soluble fms-like tyrosine kinase-1 (sFlt-1), which both derive from the placenta, are biomarkers for preeclampsia with PlGF as one of the most highly modulated maternal blood proteins during the gestational age span [12, 13]. Furthermore the ratio of PlGF/sFlt-1 is a marker for the burden of placental lesions consistent with uteroplacental underperfusion [14].

Our aim was to use these biomarkers to advance understanding of the physiological changes that occur in the maternal system after delivery.

## Methods

The study was nested within a prospective cohort study conducted at the University Hospital of Basel between 2012 and 2015 [15–17]. Following approval by the Northwest Switzerland Ethics Committee (PB_2016–02490), written informed consent was obtained from all participants. The above mentioned prospective cohort study focused on the diagnostic accuracy of biomarker cut-off values in the assessment of preeclampsia. Throughout the study a subgroup study was performed which focused on the postpartal course that resulted in the indicated study number of 56 patients with paired ante- and postpartum blood samples. Women were included when they were aged > 18 years with a singleton pregnancy and presented at least one risk factor for preeclampsia such as obesity with a body mass index (BMI) > 26.1 kg/m^2^, age > 40 years, preexisting or gestational diabetes mellitus, essential hypertension or renal disease, pregnancy-induced hypertension, uteroplacental dysfunction, previous preeclampsia, eclampsia or HELLP Exclusion criteria were chromosomal aberrations and fetal malformations, abortion, or stillbirth < 22 weeks of gestation. Demographic characteristics and medical history were recorded prospectively, and serum samples were obtained one day before and one day after parturition.

Antecubital blood samples were processed using a standardized procedure, consisting in transfer to a central laboratory, centrifugation, preparation of serum aliquots, and storage at − 80 °C until analysis. No sample had previously been thawed. Assay staff were blinded to patients’ clinical information and pregnancy outcome.

Serum sFlt-1 (pg/ml) and PlGF (pg/ml) were measured by Roche Elecsys assay on two electrochemiluminescence immunoassay platforms: Modular E170 (Roche Diagnostics, Rotkreuz, Switzerland) to October 2014 and Cobas 6000 (Roche Diagnostics) from November 2014 to study end [18]. For quality control samples the within-run coefficient of variation was below 1.5% for the sFlt-1 and below 0.9% for the PlGF assay on Modular E170. Between-run coefficients of variation were 2.5 to 3.9% for the sFlt-1 and 2.7 to 3.7% for the PlGF assay on Modular E170 and 1.2 to 2.3% for the sFlt-1 and 1.7 to 2.0% for the PlGF assay on the Cobas 6000 platform.

NfL (pg/ml) was determined by Simoa assay as previously described [19, 20].

MR-proANP (pmol/l) and copeptin (pmol/l) were measured in a single batch using fully automated BRAHMS KRYPTOR assays (B·R·A·H·M·S GmbH, part of Thermo Fisher Scientific, Hennigsdorf, Germany) [16].

Progesterone (pg/ml) was measured by ELISA kit (Enzo Life Sciences, Inc., Farmingdale, New York) according to the manufacturer’s protocol.

### Statistical analysis

Continuous variables are presented as median with interquartile range, and categorical variables as number of cases and percentages. Ante- vs postpartum biomarker changes were assessed using the non-parametric Wilcoxon matched-pairs signed rank test. Ante- and postpartum relationships between biomarkers were assessed by Spearman’s correlation and displayed in a heat map: individual coefficients (Spearman’s rho) were presented in a matrix as different color gradients, from blue (absolute positive correlation: coefficient 1) through red (absolute negative correlation: coefficient − 1). Linear regression analyses (univariable and multivariable models) explored the determinants of each biomarker change after delivery (calculated as the postpartum value/antepartum value ratio and log transformed) and was performed in two steps: a) single (exploratory) regression, in which the effect of each parameter was assessed separately, and b) multivariable modeling, in which only parameters with statistical significance *P* < 0.100 in the exploratory analysis were included in the multivariable model. Statistical analyses were performed using SPSS version 24.0 (IBM Corp., Armonk, New York; RRID:SCR_002865).

## Results

**Fig. 1:**
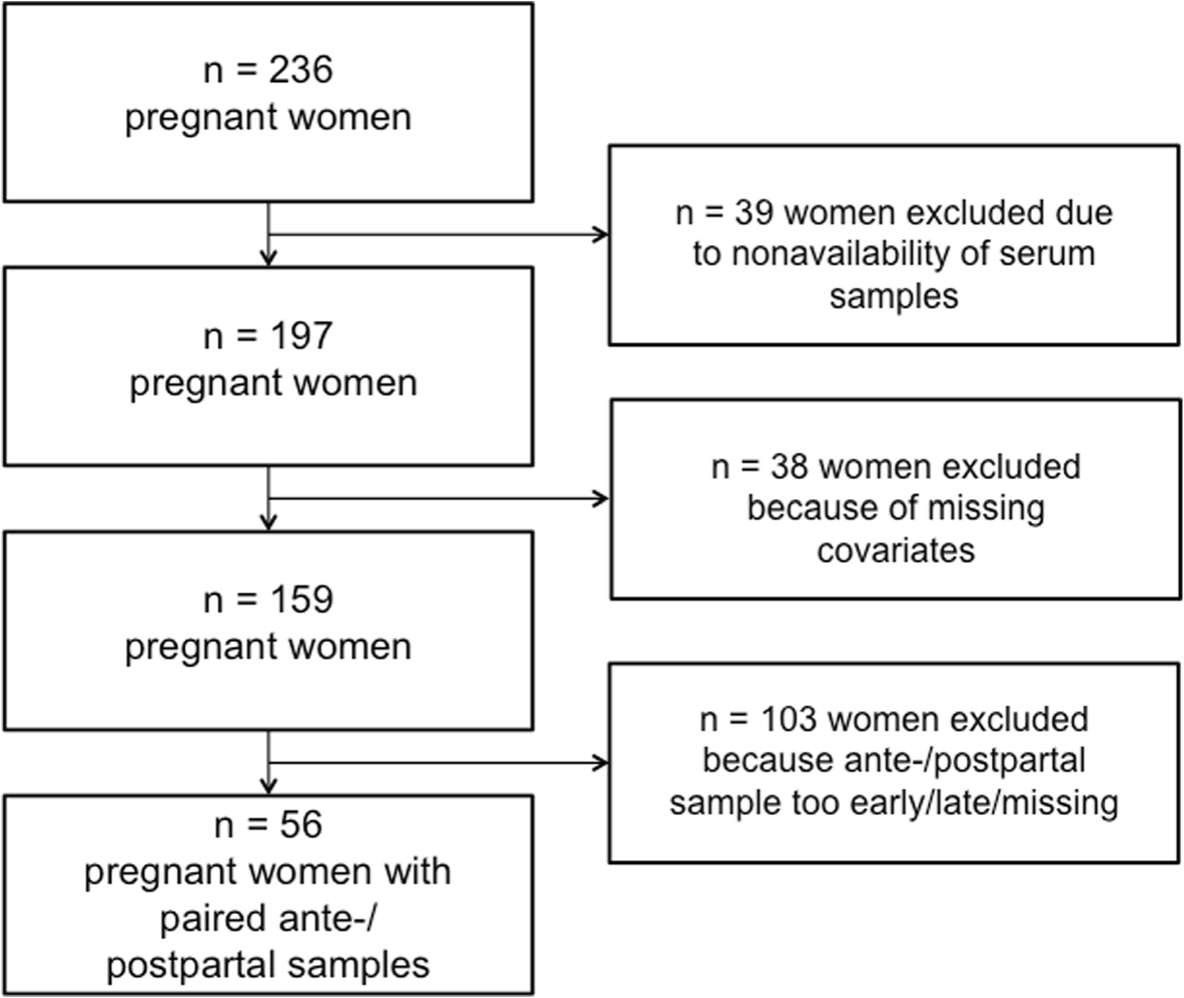
Study flow chart

**Fig. 2:**
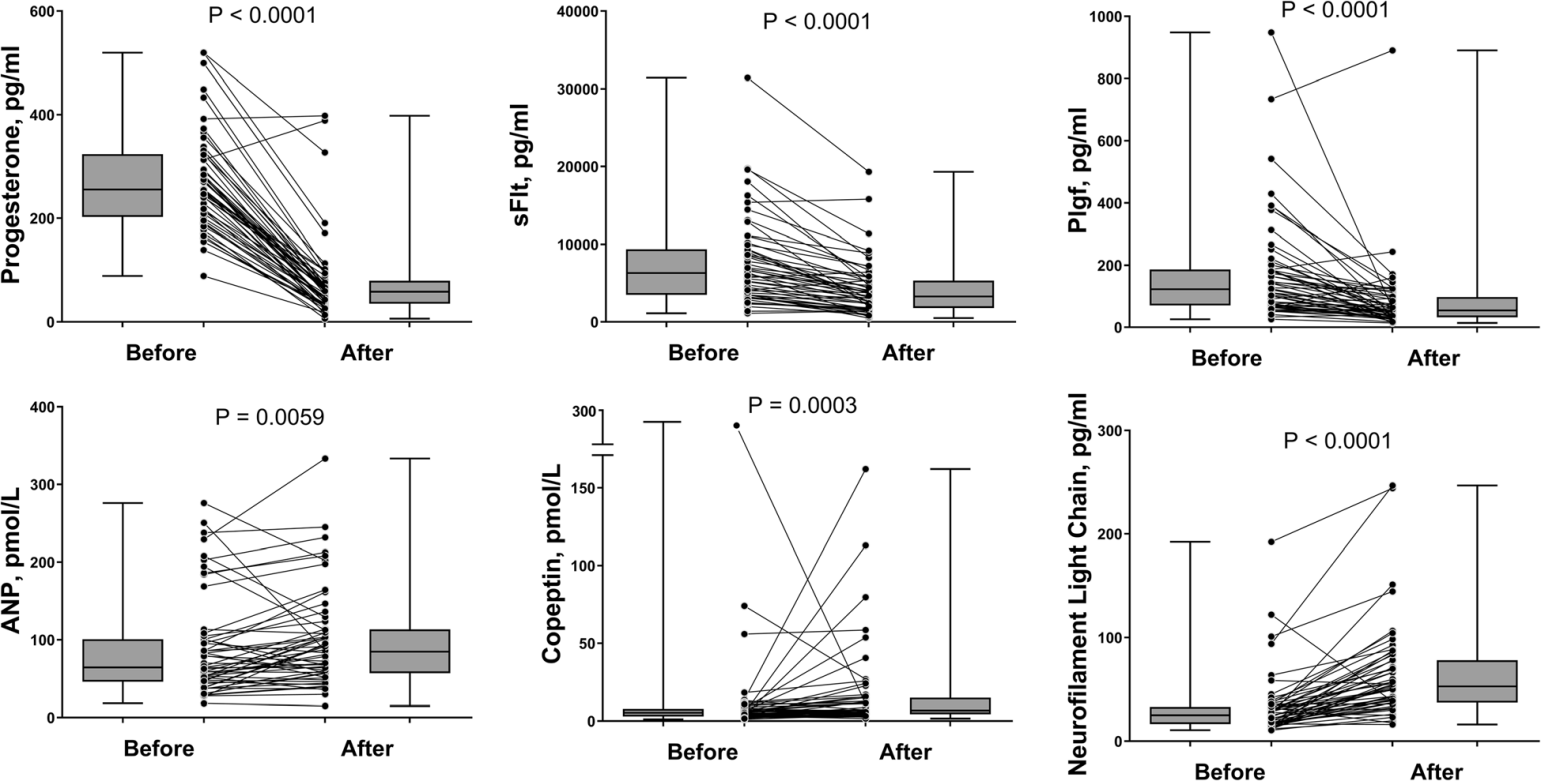
*Biomarker changes before and after delivery*. Statistical significance was assessed by the Wilcoxon matched-pairs signed rank test. sFlt-1: soluble fms-like tyrosine kinase-1, PlGF: placental growth factor, MR-proANP: by-product of atrial natriuretic peptide

**Fig. 3:**
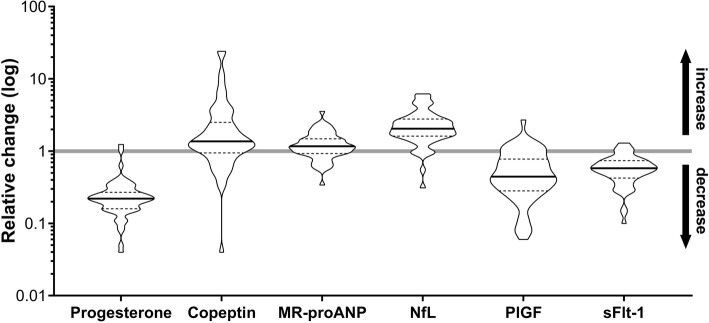
*Relative change (antepartum to postpartum ratio) of key biomarkers.* Values are presented as “violin” plots showing both the range and density of the distribution. The line of equality (no change) is also depicted

**Fig. 4:**
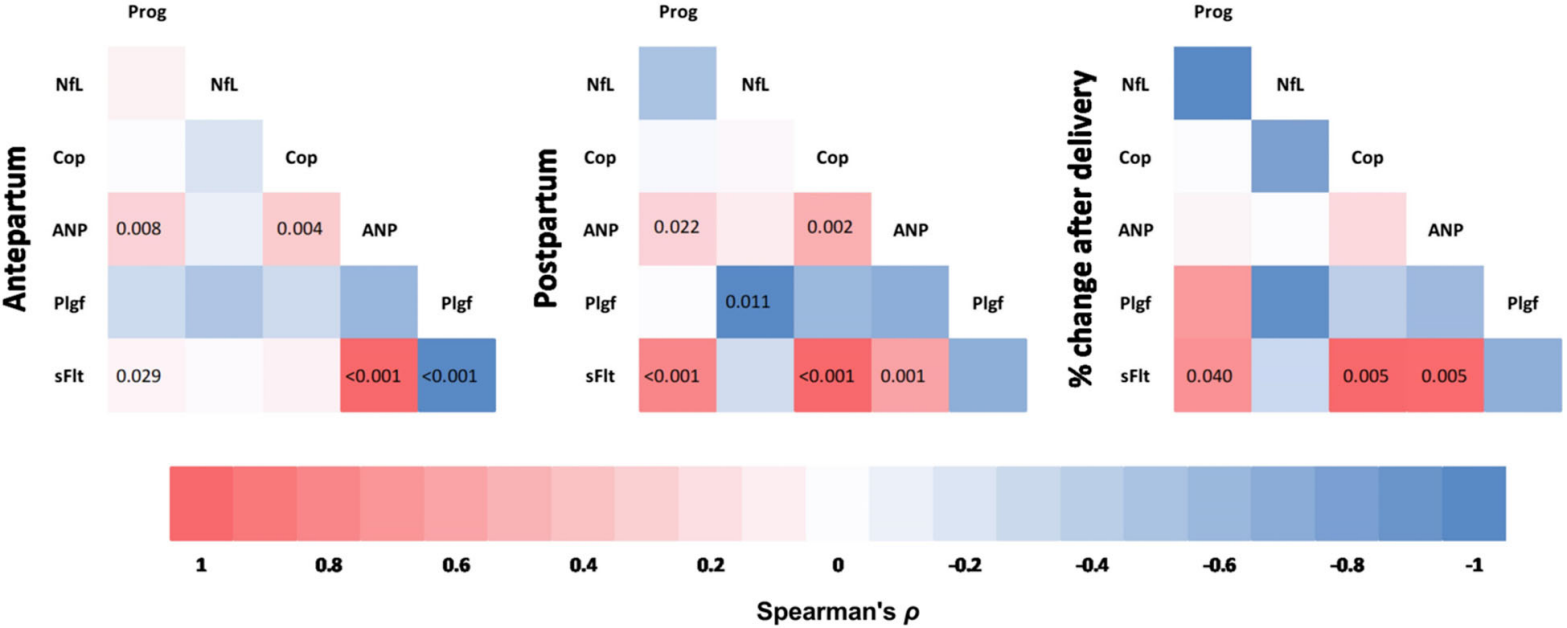
*Heat map showing the level of correlation between biomarkers before and after delivery*. Correlation coefficients (Spearman’s rho) are presented as different color gradients, from red (absolute positive correlation: correlation coefficient 1) to blue (absolute negative correlation: correlation coefficient − 1). The numbers in the cells represent the correlation *p* value (only significant *p* values are shown)

The study enrolled 56 women with paired ante- and postpartum serum samples (Fig. 1) and complete clinical and biomarker data (Table 1). Comparison between ante- and postpartum values (Table 2) showed that placental markers (progesterone, sFlt-1, and PlGF) decreased postpartum as expected, whereas cardiovascular (stress-related) biomarkers MR-proANP and copeptin, and neuronal injury marker NfL, increased significantly (Fig. 2). The relative change (prepartum to postpartum ratio) of each parameter is presented in Fig. 3 and the exact values in the supplement (Additional file 1: Table S1). The specific correlations between these biomarkers before and after delivery are presented in Fig. 4.

**Table 1:** Baseline characteristics of the study cohort

*Maternal characteristics*	
Age, years	32.6 ± 4.8 (23–45)
BMI, kg/m^2^	31.9 ± 6.4 (23.0–49.5)
Parity	
1	37 (66.1)
2+	19 (33.9)
*Gestation and delivery*	
Gestational age, weeks	38.1 ± 2.3 (32.3–41.3)
Prematurity	15 (26.8)
Gestational diabetes	17 (30.4)
- Insulin-dependent	9 (52.9)
- Lifestyle management	8 (47.1)
Preeclampsia	21 (37.5)
- Early-onset	4 (19)
- Late-onset	17 (81)
- Medical treatment	16 (76.2)
- Labetalol	16 (100)
- Methyldopa	1 (6.2)
- Nifedipine	1 (6.2)
- Magnesium	16 (100)
Delivery mode	
Vaginal	31 (55.4)
Cesarean section	25 (44.6)
- Primary	16 (28.6)
- Secondary	9 (16.1)
Epidural anesthesia	51 (91.1)
Narcotics for pain during labor	0 (0)
*Neonatal characteristics*	
Male sex	29 (51.8)
Birth weight, g	2893 ± 702 (1150–4095)
Birth length, cm	47.3 ± 3.8 (35–54)
Apgar score (5 min)	9.1 ± 1.1 (5–10)
pH (umbilical blood)	7.27 ± 0.5 (7.14–7.39)
*Blood sampling*	
Sampling during active labor	18 (32.1)
Before birth, hours	79.3 ± 21.6 (0.5–797)
After birth, hours	12.1 ± 1.9 (2.1–97)
Data are mean ± SD (range) or number of cases (%)

**Table 2:** Key ante- and postpartum parameters

	Antepartum	Postpartum	*p*-value
SBP, mmHg	145 (127–158)	142 (131–153)	0.9777
DBP, mmHg	80 (74–89)	80 (69–85)	0.4839
Hemoglobin, g/l	128 (122–132)	115 (106–125)	< 0.0001
Progesterone, pg/ml	254 (207.9–318)	59.6 (35.5–83.1)	< 0.0001
Copeptin, pmol/L	6.2 (3.4–8.3)	7.2 (5.3–15.6)	0.0003
MR-proANP, pmol/L	85.5 (58.4–141)	101.5 (65.6–154)	0.0059
NfL, pg/ml	27.7 (16.7–31.4)	49.4 (34.7–77.8)	< 0.0001
PlGF, pg/ml	113 (68.5–184)	53 (35.5–102.5)	< 0.0001
sFlt-1, pg/ml	7803 (4817–11,985)	4083 (2433–5822)	< 0.0001

Data presented as median (interquartile range). Statistical significance was assessed by Wilcoxon’s matched-pairs signed rank test

*SBP* systolic blood pressure, *DBP* diastolic blood pressure, *MR-proANP* by-product of atrial natriuretic peptide, *NfL* neurofilament light chain, *PlGF* placental growth factor, *sFlt-1* soluble fms-like tyrosine kinase-1

As anticipated our experiments demonstrated that the angiogenesis-related biomarkers sFlt-1 and PlGF showed an inverse relationship and that sFlt-1 was higher and PlGF was lower in PE, both antepartum and postpartum (sFlt-1 antepartum: 8.999 (7433–13,082) pg/ml vs. 4254 (3045–6671) pg/ml; *P* < 0.001 and sFlt-1 postpartum: 5341 (2644–7225) pg/ml vs. 3017 (1647–3834) pg/ml; *P* = 0.002; PIGF antepartum: 83 (65–142) pg/ml vs. 164 (86.5–158.5) pg/ml; *P* = 0.014; PIGF postpartum: 40 (28–60) pg/ml vs. 64 (38–107) pg/ml; *P* = 0.033). Upon closer examination of the biomarkers’ change we evaluated a sFlt-1 ratio of 0.52 (0.42–0.68) with PE vs. 0.60 (0.47–0.75) without PE (*p* = 0.268) and a PIGF ratio of 0.45 (0.29–0.97) with PE vs. 0.43 (0.29–0.68) without PE (*p* = 0.559).

Linear regression analyses exploring the determinants of postpartum biomarker change are presented as supplements (Additional file 1: Tables S2-S7). In brief, except for sFlt-1, antepartum levels were the strongest predictor of individual biomarker change. In particular, the higher the levels of progesterone and PlGF antepartum, the greater the decrease postpartum. In contrast, the lower the antepartum levels of copeptin, MR-proANP, and NfL, the greater their postpartum increase. Change in MR-proANP was also independently determined by maternal age, while that in sFlt-1 was influenced by the change in hemoglobin. In addition, the younger the mother, the lower the antepartum NfL. Although NfL increased after delivery in 49 women and decreased in the remainder, the two groups did not differ significantly in clinical characteristics (Additional file 1: Table S8). Moreover, vaginal delivery subgroup analysis showed no correlation between ante- or postpartum biomarker levels and the duration of either the first or second stage of labor (data not shown).

## Discussion

In this prospective study we showed that the progesterone and angiogenic biomarkers PlGF and sFlt-1 decrease after delivery, whereas stress marker copeptin and heart failure marker MR-proANP increase. However, the key finding was that neuronal injury marker NfL increases postpartum, independently of clinical variables or other biomarkers.

Maternal serum progesterone plunges after placental delivery [21], initiating profound endocrine adaptations, including onset of lactation [22] and reversal of the pregnancy-induced changes in the angiogenic system, with a return to non pregnant PlGF and sFlt-1 levels [23]. Negative correlation between PlGF and sFlt-1 is well documented: as term approaches the relationship becomes progressively reciprocal, with lower free PlGF levels and rising levels of total sFlt-1, especially in preeclampsia [12]. This contrasts with the positive correlation between the rising levels of progesterone and MR-proANP as gestation advances [24]. Nevertheless it is crucial to note that PE did not affect the direction of change of both sFlt-1 and PlGF – both biomarkers decreased after parturition, in a way similar to that of normotensive women.

Vaginal delivery in particular is stressful, as reflected in the postpartum increases in the stress marker copeptin and cardiac marker MR-proANP [25, 26]. However, mode of delivery was not a significant determinant of either biomarker in our cohort, perhaps due to the blood sampling time points and short biomarker half-lives (≈60 min each, measured in non-pregnant individuals) [8, 27]. In a previous study blood samples were collected around 30 min after birth in contrast to the average of 13 h after birth in our study [26]. Our group along with others previously stated, that MR-proANP can represent a supplement to the well-established biomarkers and can support diagnosis of PE at triage [16, 28]. The biomarker reflects cardiovascular hemodynamic stress, arterial stiffness and may display the severity of hypertension [11]. Despite the fact that the N-terminal pro B-type natriuretic peptide (NT-proBNP) is considered as the Gold Standard biomarker in heart failure MR-proANP emerges as a valuable biomarker for the prediction of death and heart failure related events in patients with hypertrophic cardiomyopathy and has shown similar diagnostic performance when compared with NT-proBNP [29–31]. Although MR-proANP shows significant associations with indexes of target organ damage, its ability to discriminate between normal and “abnormal” indexes of heart failure or peripheral arterial disease such as ankle-brachial index, urinary albumin creatinine ratio or left ventricular mass index is relatively modest [11]. Published data on peripartal measurement of hemodynamics are scarce due to the limited evaluation methods with rarely performed invasive methods and the difficulty of continuous monitoring with noninvasive methods [32, 33]. Nevertheless one study focused on hemodynamics immediately after vaginal delivery in healthy pregnant women and noted a significant increase in heart rate, stroke volume and cardiac output at the time of newborn delivery compared with the baseline value measured at the onset of labor with the heart rate decreasing to baseline ten minutes after birth whereas stroke volume and cardiac output decreasing but remaining higher that at labor onset until 120 min after delivery. The authors interpret this as a temporary increase in the circulating blood volume by transfusion from the uterus and/or release of inferior vena cava compression associated with uterine contractions [34]. To our knowledge the association of cardiac biomarkers and ventricular function has so far not been investigated around delivery but in pregnancies complicated by pregnancy-induced hypertension, where an impaired systolic function accompanied by an elevation of NT-proBNP levels were detected [35]. We therefore suggest that the relative change of MR-proANP of 1.28 in our study might also be due to the strain on the heart by the abovementioned increase in cardiac output occurring right after delivery.

The only identifiable factor affecting postpartum NfL was antepartum NfL, for which the sole identifiable determinant was maternal age: levels were lower in younger women. Increased NfL showed no association with either clinical characteristics or other biomarkers. We therefore speculate that the increase is triggered by parturition per se. This could be consistent with the incrimination of the long-lasting oxidative and/or psychogenic stress associated with vaginal delivery in the 2-fold increase in postpartum serum levels of glial-specific S100 calcium-binding protein B seen in women giving birth spontaneously vs those undergoing elective cesarean section [36]. Although no difference has been reported in nerve growth factor levels between pregnancy and one week postpartum [37], it should be borne in mind that nerve growth factor is a neurotrophic marker rather than a marker of neuronal damage.

Generally, acute or chronic neuroaxonal damage elevates serum NfL levels via potentially three different mechanisms: (i) neuronal destruction in the central or peripheral nervous system, (ii) increased blood-brain barrier (BBB) permeability, and (iii) increased neuronal turnover [38]. The latter is a rather unlikely explanation for the peripartum increase in NfL, but parturition may affect neuronal integrity by impairing BBB permeability. In animal studies elevated levels of vascular endothelial growth factor (VEGF) increase BBB permeability during pregnancy via complex interaction between VEGF and its two receptors but so far the role of VEGF on the BBB is not known in human pregnancy and preeclampsia [39].

In addition, we and others recently showed that NfL levels increase during pregnancy, above all in women at risk of, or with early signs of, preeclampsia [18, 40]. Recent data show focal volume reduction in gray matter in first-time mothers persisting for at least two years postpartum [41]. Whether increased postpartum NfL is associated with these structural brain changes remains to be determined.

Interestingly, postoperative NfL levels in non-pregnant surgical patients increase significantly over preoperative values, in a range very similar to the almost 2-fold increase we identified, suggesting that general anesthesia and surgery are associated with at least short-term neuronal damage [42, 43]. Given that none of our women underwent general anesthesia but 91% had epidural anesthesia and that NfL levels did not differ between women delivering spontaneously compared to those delivering by caesarean we conclude that giving birth per se has a negative impact on neuronal integrity.

We cannot exclude the possibility that in addition to the central nervous system the peripheral nervous system or other tissues also contributes to the increase in NfL. For example, the human uterus is a highly innervated muscle with abundant adrenergic and cholinergic fibers [44] and its involution after delivery may be accompanied by axon destruction. However, to the best of our knowledge, neuroaxonal injury in the postpartum uterus has not yet been reported and therefore remains speculative. Furthermore we cannot rule out that alternative sources such as the thyroid, adrenal or parathyroid gland which all feature ribonucleic acid (RNA) expression or adipose and soft tissue where NfL protein expression can be detected (data available from v19.proteinatlas.org) and which are much affected tissues during labor play a role in the increased NfL concentrations [45]. Another potential source is the fetal or placental compartment. As far as we currently know the placenta has not yet been investigated in relation to NfL. Since the molecular size of NfL has a size of around 60–70 kDa and the human placenta is freely permeable to solutes of 1350–5200 Da we do not believe that the fetus is a probable source for NfL [46–48]. Once again we also want to point out, that our cohort is a group of pregnant women at high risk to develop preeclampsia of which some also show conditions such as diabetes. To what extent these co-morbidities play a role in comparison has to be the subject of further studies.

A major limitation of our study is on the one hand the lack of blood samples from additional time points to explore the dynamics of NfL and other biomarkers over a longer period before and after delivery and on the other hand the absence of CSF samples to be able to validate the intracerebral origin of NfL.

## Conclusions

In summary, our study characterizes the evolution of various cardiovascular and neuronal serum biomarkers from before to after parturition in a cohort at high risk of developing preeclampsia and shows for the first time that maternal serum NfL levels increase postpartum independently of delivery mode, gestational age and other clinical parameters. Additional studies are needed to verify the hypothesis that parturition per se has an impact on maternal neuronal integrity.

## Supplementary information

**Additional file 1: Table S1.** Relative antenatal - postnatal change in key parameters**. Table S2.** Relationships of Copeptin change (log) after delivery. **Table S3.** Relationships of MR-proANP change (log) after delivery. **Table S4.** Relationships of NfL change (log) after delivery. **Table S5.** Relationships of PlGF change (log) after delivery **Table S6.** Relationships of sFlt-1 change (log) after delivery. **Table S7.** Determinants of NfL levels (log) before delivery. **Table S8.** Comparisons between cases in which NfL increased and those in which NfL decreased after delivery.

## Data Availability

The datasets used and analysed during the current study are available from the corresponding author on reasonable request.

## Abbreviations

BBB: Blood-brain barrier
BMI: Body mass index
CNS: Central nervous system
CSF: Cerebrospinal fluid
CT-proAVP: C-terminal pro-arginine vasopressin
ELISA: Enzyme-linked immunosorbent assay
HELLP: Acronym for hemolysis-elevated liver enzymes low platelet count
kDa: Kilodalton
MR-proANP: Mid-regional pro-atrial natriuretic peptide
Nf: Neurofilaments
NfH: Neurofilament heavy chain
NfL: Neurofilament light chain
NT-proBNP: N-terminal pro B-type natriuretic peptide
pg/ml: Picograms per millilitre
PlGF: Placental growth factor
pmol/l: Picomoles per litre
RNA: Ribonucleic acid
RRID: Research Resource Identifiers
SD: Standard deviation
sFlt-1: Soluble fms-like tyrosine kinase-1
Simoa: Single molecule array
VEGF: Vascular endothelial growth factor
