# Supplementary material for: Impact of parturition on maternal cardiovascular and neuronal integrity in a high risk cohort – a prospective cohort study
Source: BMC Pregnancy Childbirth. 2019 Nov 5;19:403. doi: 10.1186/s12884-019-2570-6 (PMC6833198; doi:10.1186/s12884-019-2570-6)
Supplement: Supplementary file 1 — Additional file 1: Table S1. Relative antenatal - postnatal change in key parameters. Table S2. Relationships of Copeptin change (log) after delivery. Table S3. Relationships of MR-proANP change (log) after delivery. Table S4. Relationships of NfL change (log) after delivery. Table S5. Relationships of PlGF change (log) after delivery Table S6. Relationships of sFlt-1 change (log) after delivery. Table S7. Determinants of NfL levels (log) before delivery. Table S8. Comparisons between cases in which NfL increased and those in which NfL decreased after delivery. [file 12884_2019_2570_MOESM1_ESM.docx]

**Supplemental Tables**

| **Table S1.** Relative antenatal - postnatal change in key parameters | |
| --- | --- |
|  | Relative change* |
| SBP, mmHg | 0.99 (0.92 - 1.07) |
| DBP, mmHg | 0.98 (0.85 - 1.10) |
| Hemoglobin, g/l | 0.89 (0.83 - 0.98) |
| Progesterone, pg/ml | 0.22 (0.16 - 0.27) |
| Copeptin, pmol/L | 1.35 (0.85 - 2.98) |
| MR-proANP, pmol/L | 1.14 (0.94 - 1.46) |
| NfL, pg/ml | 2.07 (1.61 - 2.62) |
| PlGF, pg/ml | 0.46 (0.31 - 0.98) |
| sFlt-1, pg/ml | 0.58 (0.37 - 0.70) |
| Data presented as median (interquartile range).  * calculated as the antepartum to postpartum ratio; values >1 represent an increase and values <1 a decrease in the postpartum parameter  SBP: systolic blood pressure, DBP: diastolic blood pressure, MR-proANP: by-product of atrial natriuretic peptide, NfL: neurofilament light chain, PlGF: placental growth factor, sFlt-1: soluble fms-like tyrosine kinase-1. | |

| **Table S2. Relationships of Copeptin change (log) after delivery** | | | | |
| --- | --- | --- | --- | --- |
|  | Univariable models | | Multivariable model  (R^2^ 0.414) | |
|  | beta | p-value | beta | p-value |
| Maternal age | 0.236^a^ | 0.080 | 0.123 | 0.390 |
| Maternal BMI | 0.110 | 0.429 |  |  |
| Parity | –0.080 | 0.556 |  |  |
| GDM | 0.159 | 0.242 |  |  |
| PE | 0.284^b^ | 0.034 | 0.213 | 0.121 |
| CS | –0.127 | 0.352 |  |  |
| GA | –0.336^c^ | 0.011 | –0.124 | 0.377 |
| Anesthesia | –0.137 | 0.315 |  |  |
| Male sex | 0.109 | 0.422 |  |  |
| Hb change | 0.397^d^ | 0.016 | 0.198 | 0.191 |
| SBP change | –0.038 | 0.795 |  |  |
| DBP change | 0.077 | 0.605 |  |  |
| Cop before | –0.523^e^ | <0.001 | –0.433 | 0.005 |
| Prog change | –0.046 | 0.737 |  |  |
| ANP change | 0.174 | 0.199 |  |  |
| NfL change | –0.108 | 0.429 |  |  |
| PlGF change | –0.118 | 0.386 |  |  |
| sFlt-1 change | 0.232^f^ | 0.085 | 0.111 | 0.499 |
| Only variables with significance <0.010 were introduced in the multivariable model  Coefficients of determination (R^2^): ^a^ 0.038; ^b^ 0.064; ^c^ 0.097;  ^d^ 0.133; ^e^ 0.260; ^f^ 0.036 | | | | |

| **Table S3. Relationships of MR-proANP change (log) after delivery** | | | | |
| --- | --- | --- | --- | --- |
|  | Univariable models | | Multivariable model  (R^2^ 0.291) | |
|  | beta | p-value | beta | p-value |
| Maternal age | 0.345^a^ | 0.009 | 0.268 | 0.026 |
| Maternal BMI | –0.012 | 0.933 |  |  |
| Parity | 0.145 | 0.288 |  |  |
| GDM | 0.260^b^ | 0.053 | 0.114 | 0.363 |
| PE | –0.087 | 0.526 |  |  |
| CS | –0.153 | 0.260 |  |  |
| GA | 0.007 | 0.960 |  |  |
| Anesthesia | 0.064 | 0.640 |  |  |
| Male sex | –0.121 | 0.376 |  |  |
| Hb change | 0.233 | 0.171 |  |  |
| SBP change | 0.080 | 0.589 |  |  |
| DBP change | 0.142 | 0.336 |  |  |
| ANP before | –0.455^c^ | <0.001 | –0.345 | 0.006 |
| Prog change | 0.034 | 0.806 |  |  |
| Cop change | 0.066 | 0.628 |  |  |
| NfL change | 0.029 | 0.833 |  |  |
| PlGF change | –0.180 | 0.185 |  |  |
| sFlt-1 change | 0.310^d^ | 0.020 | 0.205 | 0.092 |
| Only variables with significance <0.010 were introduced in the multivariable model  Coefficients of determination (R^2^): ^a^ 0.103; ^b^ 0.051; ^c^ 0.192;  ^d^ 0.079 | | | | |

| **Table S4. Relationships of NfL change (log) after delivery** | | | | |
| --- | --- | --- | --- | --- |
|  | Univariable models | | Multivariable model  (R^2^ 0.236) | |
|  | beta | p-value | beta | p-value |
| Maternal age | –0.074 | 0.587 |  |  |
| Maternal BMI | 0.027 | 0.846 |  |  |
| Parity | 0.091 | 0.504 |  |  |
| GDM | 0.043 | 0.753 |  |  |
| PE | –0.139 | 0.508 |  |  |
| CS | 0.044 | 0.745 |  |  |
| GA | –0.111 | 0.417 |  |  |
| Anesthesia | 0.021 | 0.885 |  |  |
| Male sex | 0.045 | 0.742 |  |  |
| Hb change | 0.249 | 0.144 |  |  |
| SBP change | –0.239 | 0.102 |  |  |
| DBP change | –0.216 | 0.141 |  |  |
| NfL before | –0.432^a^ | 0.001 | –0.424 | 0.001 |
| Prog change | –0.237^b^ | 0.078 | –0.222 | 0.070 |
| Cop change | 0.048 | 0.727 |  |  |
| ANP change | –0.011 | 0.935 |  |  |
| PlGF change | –0.105 | 0.441 |  |  |
| sFlt-1 change | –0.002 | 0.986 |  |  |
| Only variables with significance <0.010 were introduced in the multivariable model  Coefficients of determination (R^2^): ^a^ 0.171; ^b^ 0.039 | | | | |

| **Table S5. Relationships of PlGF change (log) after delivery** | | | | |
| --- | --- | --- | --- | --- |
|  | Univariable models | | Multivariable model  (R^2^ 0.221) | |
|  | beta | p-value | beta | p-value |
| Maternal age | –0.011 | 0.935 |  |  |
| Maternal BMI | 0.086 | 0.536 |  |  |
| Parity | 0.054 | 0.694 |  |  |
| GDM | –0.160 | 0.238 |  |  |
| PE | 0.090 | 0.510 |  |  |
| CS | –0.091 | 0.504 |  |  |
| GA | –0.118 | 0.386 |  |  |
| Anesthesia | 0.128 | 0.349 |  |  |
| Male sex | 0.178 | 0.189 |  |  |
| Hb change | –0.192 | 0.262 |  |  |
| SBP change | 0.226 | 0.123 |  |  |
| DBP change | 0.043 | 0.773 |  |  |
| PlGF before | –0.446^a^ | 0.001 | –0.427 | 0.001 |
| Prog change | 0.261^b^ | 0.052 | 0.226 | 0.064 |
| Cop change | –0.008 | 0.955 |  |  |
| ANP change | –0.105 | 0.443 |  |  |
| NfL change | –0.204 | 0.132 |  |  |
| sFlt-1 change | –0.114 | 0.405 |  |  |
| Only variables with significance <0.010 were introduced in the multivariable model  Coefficients of determination (R^2^): ^a^ 0.184; ^b^ 0.051 | | | | |

| **Table S6. Relationships of sFlt-1 change (log) after delivery** | | | | |
| --- | --- | --- | --- | --- |
|  | Univariable models | | Multivariable model  (R^2^ 0.433) | |
|  | beta | p-value | beta | p-value |
| Maternal age | 0.128 | 0.345 |  |  |
| Maternal BMI | –0.095 | 0.493 |  |  |
| Parity | 0.125 | 0.360 |  |  |
| GDM | 0.262^a^ | 0.051 | 0.256 | 0.114 |
| PE | –0.114 | 0.401 |  |  |
| CS | 0.009 | 0.948 |  |  |
| GA | 0.031 | 0.822 |  |  |
| Anesthesia | –0.133 | 0.330 |  |  |
| Male sex | 0.074 | 0.587 |  |  |
| Hb change | 0.479^b^ | 0.003 | 0.491 | 0.003 |
| SBP change | 0.233 | 0.111 |  |  |
| DBP change | 0.193^c^ | 0.082 | 0.158 | 0.288 |
| sFlt-1 before | –0.148 | 0.276 |  |  |
| Prog change | 0.244^d^ | 0.070 | 0.487 | 0.003 |
| Cop change | 0.066 | 0.631 |  |  |
| ANP change | 0.259^e^ | 0.054 | –0.011 | 0.944 |
| NfL change | –0.021 | 0.879 |  |  |
| PlGF change | –0.202 | 0.135 |  |  |
| Only variables with significance <0.010 were introduced in the multivariable model  Coefficients of determination (R^2^): ^a^ 0.051; ^b^ 0.206; ^b^ 0.044; ^d^ 0.042; ^e^ 0.050 | | | | |

| **Table S7. Determinants of NfL levels (log) before delivery** | | | | |
| --- | --- | --- | --- | --- |
|  | Univariable models | | Multivariable model  (R^2^ 0.132) | |
|  | beta | p-value | beta | p-value |
| Maternal age | 0.332^a^ | 0.012 | 0.272 | 0.043 |
| Maternal BMI | 0.047 | 0.733 |  |  |
| Parity | -0.190 | 0.161 |  |  |
| GDM | -0.070 | 0.605 |  |  |
| PE | 0.237^b^ | 0.078 | 0.214 | 0.186 |
| GA | -0.135 | 0.320 |  |  |
| SBP before | 0.242^c^ | 0.084 | 0.079 | 0.622 |
| DBP before | 0.214 | 0.107 |  |  |
| Hb before | -0.084 | 0.560 |  |  |
| Prog before | 0.228^d^ | 0.091 | 0.185 | 0.168 |
| Cop before | -0.016 | 0.908 |  |  |
| ANP before | 0.097 | 0.475 |  |  |
| PlGF before | -0.143 | 0.292 |  |  |
| sFlt-1 before | 0.100 | 0.463 |  |  |
| Only variables with significance <0.10 were introduced in the multivariable model  Coefficients of determination (R^2^): ^a^ 0.094; ^b^ 0.039; ^c^ 0.040;  ^d^ 0.034; | | | | |

| **Table S8.** Comparisons between cases in which NfL increased and those in which NfL decreased after delivery | | | |
| --- | --- | --- | --- |
|  | **NfL increase (n=49)** | **NfL decrease (n=7)** | **P value*** |
| Maternal age, years | 32.8 ± 5.1 | 31.1 ± 2.7 | 0.422 |
| Maternal BMI, kg/m^2^ | 32.1 ± 6.2 | 30.6 ± 8.5 | 0.286 |
| Parity |  |  |  |
| 1 | 33 (67.3) | 4 (57.1) | 0.242 |
| 2 | 7 (14.3) | 3 (42.9) |  |
| 3 | 6 (12.2) | 0 |  |
| 4 | 3 (6.1) | 0 |  |
| Gestational diabetes | 14 (28.6) | 3 (42.9) | 0.662 |
| Preeclampsia | 17 (34.7) | 4 (57.1) | 0.406 |
| Cesarean section | 23 (46.9) | 2 (28.6) | 0.443 |
| Epidural anesthesia | 44 (89.8) | 7 (100) | 0.499 |
| Gestational age, weeks | 38.1 ± 2.5 | 38.4 ± 1.1 | 0.758 |
| Male sex | 26 (53.1) | 3 (42.9) | 0.700 |
| pH (umbilical blood) | 7.27 ± 0.5 | 7.27 ± 0.6 | 0.947 |
| SBP change | 1.00 ± 0.15 | 1.02 ± 0.09 | 0.732 |
| DBP change | 0.98 ± 0.17 | 1.00 ± 0.20 | 0.698 |
| Hb change | 0.91 ± 0.10 | 0.85 ± 0.08 | 0.161 |
| Antepartum NfL levels, pg/ml | 30.6 ± 29.6 | 46.3 ± 35.7 | 0.052 |
| Data are mean ± SD (range) or number of cases (%) | | | |
